# Editorial: Applied and sustainable methods to manage environmental contaminants with natural and fortified microbial biosorbents

**DOI:** 10.3389/fmicb.2023.1220337

**Published:** 2023-06-07

**Authors:** Surojit Bera, Debabrata Chowdhury, Jing Han, Joginder Singh

**Affiliations:** ^1^Department of Microbiology, School of Bioengineering and Biosciences, Lovely Professional University, Phagwara, Punjab, India; ^2^School of Medicine - Infectious Diseases, Stanford University, Stanford, CA, United States; ^3^State Key Laboratory of Microbial Resources, Institute of Microbiology, Chinese Academy of Sciences, Beijing, China; ^4^College of Life Science, University of Chinese Academy of Sciences, Beijing, China

**Keywords:** microbial biomass, biosorbent, contaminants, environmental pollution, biochar

Population explosion and the industrial revolution are building a massive but indiscriminate and inappropriate use of energy and natural resources, ultimately leading to objectionable environmental pollution and subsequent health problems. The existence of these diverse pollutants, such as heavy metals, synthetic colorants, plastics and microplastics, biomedical wastes, toxic agrochemicals (pesticide, herbicide and fertilizer), also is imposing unbearable physiological threats for aquatic lives *via* bioaccumulation and biomagnification (Bera et al., 2016; Sidhu et al., 2019; Singh et al., 2021; Saravanan et al., 2022; Yadav et al., 2022; Thakur et al., 2023). Removal or management of these pollutants primarily depends upon the chemical nature of contaminants, place of application (*ex-situ* or *in-situ*), functioning conditions (like oxygen and nutrient concentrations, temperature, pH, and other abiotic factors), cost-effectiveness, environmental policies etc., while electing any bioremediation techniques (Medfu Tarekegn et al., 2020). With this background and magnitude, currently, the biomass of biological agents such as algae, bacteria, fungi or yeast are being used by researchers as an efficient contaminant remover naturally or as in fortified form, thus making the remediation process eco-friendly, cost-effective, and scalable (Bhatia et al., 2017).

Under this current Research Topic, we have received studies and findings from 43 different researchers out of seven intercontinental countries who have addressed the environmental pollution issue as a genuine threat to the globe. Additionally, their work has revealed the hidden potential of microbe's natural or fortified biomass to deal with such contaminants. Dusengemungu et al. investigated the potential of several autochthonous filamentous fungi isolated from the heavy metal-contaminated soil of Zambia. Being a developing country, Zambian soils are less established as a potential reservoir for microbial diversity that can take part in active bioremediation, even though soil pollutants like heavy metal contamination are evident in Zambian provinces. “Nkana Slag Dumpsite”—the “Black Mountain” and Uchi Tailing Dam (TD26) of Kitwe District were chosen (also referred to as Copperbelt province) by them for isolation of three indigenous filamentous fungi, namely *Aspergillus transmontanensis, Cladosporium cladosporioides*, and *Geotrichum candidum* spp. capable of reducing Cu, Co, Zn, Fe, Mn and Pb from heavy metal-contaminated soil. After doing a paired interaction compatibility study in standard fungal media, it was ascertained that this fungus could be used as a consortium in bioaugmentation for *in-situ* bioremediation of heavy metal-contaminated soil. Another independent study by Cao et al. also showed that their isolated *Pseudoalteromonas agarivorans* Hao 2018, a marine bacterium capable of producing exopolysaccharide, could be a game changer in case of lead (Pb) immobilization, thus proposing *in-situ* bioremediation of heavy metal contaminated soil. Introduction of Hao 2018 slashed Pb content in rhizospheric soil and its bioaccumulation in pakchoi (*Brassica chinensis* L.), thus leading to an increase in plant biomass, ascorbic acid and soluble protein content. Further analysis of biochemical parameters for rhizospheric soil, *viz*. pH, nitrogen content (NH${}_{4}^{+}$ and NO${}_{3}^{-}$), and soil enzymes (soil dehydrogenase activity, soil alkaline phosphatase and urease activity) indicated that reinforcement in these indexes might be responsible for the bioremediation potential of Hao 2018. Additionally, this strain amended the richness of *Streptomyces* and *Sphingomonas*, which are claimed to have a positive effect on rhizospheric soil for pakchoi. However, they have also suggested that field trial in heavy metal contaminated sites is necessary to establish these findings for large-scale application. Mehanni et al. analyzed wastewater samples contaminated with hospital discharge in Beni Suef, Egypt. They found abundant multidrug-resistant bacteria like *Escherichia coli, Enterococcus faecalis* and *Staphylococcus haemolyticus*. Though they are resistant to popular antibiotics such as Tetracycline, Ampicillin, Amoxicillin, Chloramphenicol, and Erythromycin to a certain extent, they showed sensitivity above the concentration of 50 ppm. Their controlled pots experiment setup in the greenhouse conditions at National Research Centre, Egypt, using the commercial zucchini plant (H5N5) revealed that there were no significant variances in zucchini plant biomass using freshwater or the treated hospital effluent water, although the authors underlined that the jeopardy of contaminants like fragments of cell-free DNA might persist in soil for a long time and adept to transform competent cells in the natural environment. Boruah et al. and Tripathi et al. made two critical reviews to exploit the bioremediation potential of microbial sorbents *via* unique approaches. The former study dealt with a comprehensive literature review on the practice of Fe-nanomaterials use for arsenic removal. However, they indicated some key bottleneck areas like regeneration and reusability test for adsorbents, leaching test, hydrodynamic stability, biocompatibility and toxicity study, which need proper attention to maximize the bioremediation potential for those nanocomposites. The later study successfully investigated the bioremediation potential of nanocomposites made by green approaches like microbial-assisted nanomaterials, genetic engineering, or biofilm-based nanomaterial to handle the challenges with wastewater discharge, which contains a blend of dyes or heavy metals. This review demonstrates how innovative nanocomposite and gene manipulation techniques have been used in the past decade while correlating their mode of action to achieve new directions leading to sustainable bioremediation strategies. These green technologies are promising to remove cadmium, lead, chromium, uranium, zinc, nickel, copper, arsenic and many more. However, they also indicated that optimization of physicochemical parameters such as temperature, pH, and pollutant concentration is a prerequisite for attaining ideal biosorbent performance.

This Research Topic has explored the multidimensional properties of microbial biomass in the field of environmental pollution research, making it more economically feasible and scalable. Additionally, the green technology used by the researchers here will certainly reduce the carbon footprint for the chemical procedures—which are one of the major causes of greenhouse gas emissions leading to an increase in global warming. These multidisciplinary research findings shall surely redirect the comprehensive arena of unusual challenges faced by environmentalists; also, it will be a meaningful contribution to the field of microbial bioremediation and is of interest to the broad readership of Frontiers in Microbiology.

## Author contributions

JS and JH acted as topic editors and actively look after the scope and contents of the submitted manuscripts in this issue. SB and DC acted as topic coordinators actively and supported the topic editors to proceed completion of the Research Topic. All authors contributed to the article and approved the submitted version.
